# The long-term consequences of Corona Virus Disease 2019 patients receiving Chinese herbal medicine treatments in acute phase: A protocol for systematic review and meta-analysis

**DOI:** 10.1097/MD.0000000000026677

**Published:** 2021-07-23

**Authors:** Yi-ming Sun, Jia-yan Liu, Ran Sun, Jie Zhang, Meng-lu Xiao, Gui-ping Li

**Affiliations:** a Chengdu Eighth People's Hospital (Geriatric Hospital of Chengdu Medical College), Chengdu, China; b Tianjin Xiangyang Road Community Health Service Center, China; c Chengdu University of Traditional Chinese Medicine, Chengdu, China; d First Teaching Hospital of Tianjin University of Traditional Chinese Medicine, Tianjin, China.

**Keywords:** Chinese herbal medicine, Corona Virus Disease 2019, long-term, meta-analysis, protocol, severe acute respiratory syndrome coronavirus-2, systematic review

## Abstract

**Background:**

In December 2019, the first case of Corona Virus Disease 2019 (COVID-19) associated with severe acute respiratory syndrome coronavirus-2 viral infection was described in Wuhan. Similar to SARS in 2003, COVID-19 also had a lasting impact. Approximately 76% of patients discharged after hospitalization for COVID-19 had neurological manifestations which could persist for 6 months, and some long-term consequences such as the gradual loss of lung function due to pulmonary interstitial fibrosis could have comprehensive effects on daily quality of life for people who were initially believed to have recovered from COVID-19.

**Methods and analysis:**

Our comprehensive search strategy developed in consultation with a research librarian. We will search these following electronic databases: PubMed, Cochrane Library, Web of Science, ScienceDirect, Scopus, Google Scholar, Embase, ProQuest, China Science and Technology Journal Database (VIP), China National Knowledge Infrastructure, WANFANG DATA, WHO covid-19 website, and Centers for Disease Control and the Prevention COVID-19 websites of the United States and China. The bias of publication will be confirmed via the *P* value of Egger test. The quality of studies will be evaluated by the Newcastle-Ottawa Scale.

**Ethics and dissemination:**

There are no ethical considerations associated with this study protocol for this systematic review which mainly focuses on the examination of secondary data. On completion of this analysis, we will prepare a manuscript for publication in a peer-reviewed medical journal.

**PROSPERO registration number:**

CRD42021258711.

## 1 Introduction

Corona Virus Disease 2019 (COVID-19) is caused by severe acute respiratory syndrome coronavirus-2 (SARS-COV-2). Globally, as of June 1, 2021, there have been 170,426,245 confirmed cases of COVID-19, including 3,548,628 deaths, reported to the WHO. Judging from current researches, the SARS-CoV-2 infects the upper and lower respiratory tracts, causing cough, fever, shortness of breath, and sometimes severe pulmonary symptoms, also the severity of lung damage is closely related to the severity of infection.^[1–3]^ The long-term consequences, such as the gradual loss of lung function due to pulmonary interstitial fibrosis could have adverse effects on daily quality of life for people who initially believed to have recovered from COVID-19.^[1–3]^ Furthermore, SARS-CoV-2 also acts multisystematically with substantial impact on the nervous system.^[4–11]^ Many studies about COVID-19 related diverse neurological manifestations like headache, dizziness, anosmia, and cerebrovascular events have also been frequently reported.^[10–19]^ The central nervous system and the peripheral nervous system are both acutely damaged by SARS-CoV-2 which also causes long-term damage,^[3]^ and the damage to the central nervous system following COVID-19 may be permanent.

The results have been reported from the follow-up of patients who have recovered from SARS-CoV-1 infection in 2003 could be used to indicate what to expect in the long term from SARS-CoV-2 infection.^[20,21]^ Some studies have shown that lung ventilation function in all follow-up patients with SARS-CoV-1 had varying degrees of damage, and lung diffusion function in more than one-third of patients was significantly impaired.^[20–22]^ Following SARS-CoV-2 infection, which is similar to the effects reported from SARS-CoV-1 infection, the formation of intra-alveolar thrombosis and airway inflammatory viral damage further contributes to the development of pulmonary fibrosis.^[23–25]^ Some findings support that SARS-CoV-2 can alter the blood–brain barrier to enter the brain, and support the appearance of neurological symptoms, the formation of fatal microthrombi, and even the occurrence of encephalitis associated with COVID-19.^[26,27]^ These neurologic associations of COVID-19 support the clinical reports of early neurological changes and the potential basis for the occurrence of long-term neurological sequelae.

As the initial outbreak area, China has taken multiple active measures to deal with COVID-19, one of which is the Chinese herbal medicine (CHM). CHM has played an important role in combating pandemic and endemic diseases for thousands of years. During the outbreak of severe acute respiratory syndrome (SARS) coronavirus in late 2002 in Guangdong, China, traditional CHM had showed beneficial effects such as relief of symptoms, decreased mortality, and control of infection in patients with SARS.^[28–31]^ With accumulated experiences of its anti-viral properties for the treatment of SARS and strong encouragement from the Chinese government,^[32]^ CHM has also been applied in treating COVID-19. The National Health Commission of China established the third version of National COVID-19 Diagnosis and Treatment Guideline on January 23, 2020. CHM has been recommended as a treatment for COVID-19 based on different stages and syndrome differentiation of the disease. Clinical evidence showed that compared with the treatment of Western medicine, the integration of CHM and Western medicine for COVID-19 may have better effects.^[33]^ CHM is believed to have a wide range of antiviral effects and reduce pulmonary inflammatory effects.^[34]^ Some studies have shown that CHM might have compounds with capacity against COVID-19,^[35]^ and have a role in the treatment and symptomatic management of patients with COVID-19.^[36,37]^ For example, Lianhua Qingwen capsules significantly inhibited SARS-COV-2 replication, affected virus morphology and exerted anti-inflammatory activity in vitro, and Maxing Shigan decoction significantly reduced pulmonary inflammation in vivo.

There is currently a lack of long-term observation of COVID-19 patients receiving CHM treatment in the acute phase, but it is important to make early predictions of the possible long-term sequelae of COVID-19. In this review, we will provide robust evidence for the effect of CHM on the long-term consequences of COVID-19, and suggest that CHM could be an essential alternative strategy to combat these awful viruses.

## 2 Methods

### 2.1 Study registration

The protocol was developed in accordance with the Preferred Reporting Items for Systematic Review and Meta-Analysis Protocol (PRISMA-P) guidelines. The PRISMA-P checklist was used to assess adherence to the guidelines. Categories that do not apply are marked as nonapplicable (N/A). This systematic review was registered with PROSPERO (CRD42021258711) (June 3, 2021).

### 2.2 Eligibility criteria

We will include studies on humans, which are randomized controlled trials (RCTs), prospective or retrospective case series, or cohort studies with a control arm. Only COVID-19 patients who had received Chinese herbal medicine during their hospitalization will be considered in this study. Participants in the treatment group should be treated with CHM alone or in combination with conventional pharmacotherapy. There will be no restriction on dosage, but the route of administration is oral. To ensure that all relevant articles are included, we will not set any specifications for age or ethnic origin.

### 2.3 Search strategy

A comprehensive search of bibliographic and grey literature sources, including PubMed, Cochrane Library, Web of Science, ScienceDirect, Scopus, Google Scholar, Embase, ProQuest, China Science and Technology Journal Database (VIP), China National Knowledge Infrastructure, WANFANG DATA, WHO covid-19 website, and Centers for Disease Control and Prevention COVID-19 websites of the United States and China will be performed as of June 20, 2021 without language restrictions. We will also scrutinize the bibliographies of the eligible studies and relevant review articles. The Medical Subject Headings (MeSH), free text and relevant, terms of COVID-19, and Chinese herbal medicine will be applied in our search strategy. The main keywords are COVID-2019 pneumonia, traditional Chinese medicine, sequelae, and their relevant words. The search strategy for the PubMed database is presented in Table 1.

**Table 1:** Search strategy for PubMed.

#1	COVID-19 OR SARS-CoV-2 [MeSH Terms]
#2	COVID 19 OR Covid 2019 OR COVID19 [Title/Abstract]
#3	SARS-CoV2 OR SARS CoV-2 OR SARSCoV2 OR SARSCoV-2 [Title/Abstract]
#4	2019 nCoV OR nCoV 2019 OR 2019-ncov OR ncov19 OR ncov-19 OR 2019-novel CoV [Title/Abstract]
#5	(new OR novel OR nouveau) AND (corona virus^∗^ OR coronavirus^∗^) [Title/Abstract]
#6	(Huanan OR Hubei OR Wuhan) AND (coronavirus^∗^ OR corona virus^∗^) [Title/Abstract]
#7	(SARS-coronavirus-2 OR Sars-coronavirus2 OR SARS-like coronavirus) [Title/Abstract]
#8	Severe Acute Respiratory Syndrome Coronavirus-2 OR severe acute respiratory syndrome coronavirus 2 OR Wuhan Seafood Market Pneumonia Virus [Title/Abstract]
#9	#1 OR #2 OR #3 OR #4 OR #5 OR #6 OR #7 OR #8
#10	Chinese Herbal OR traditional Chinese medicine OR Drugs [MeSH Terms]
#11	Chinese herb^∗^[Title/Abstract]
#12	Chinese medicine^∗^[Title/Abstract]
#13	TCM OR CHM [Title/Abstract]
#14	#10 OR #11 OR #12 OR #13
#15	#9 AND #14

### 2.4 Study records

#### 2.4.1 Study selection

**Figure 1:**
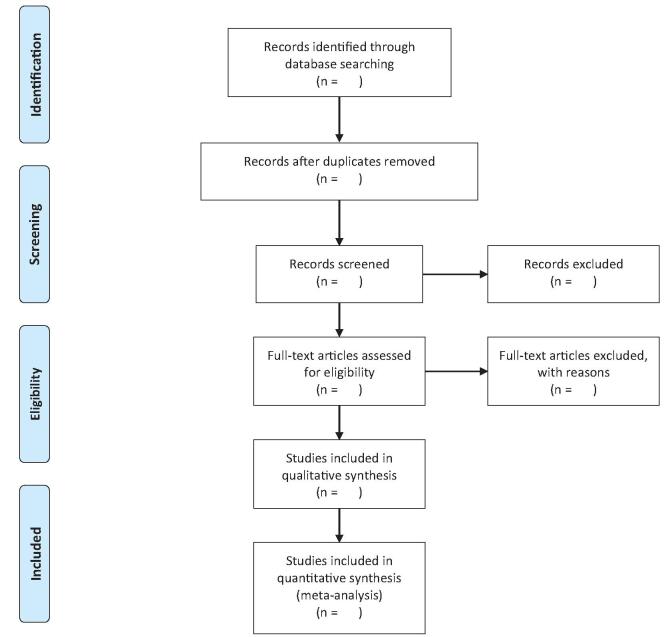
PRISMA flow chart.

The references obtained during the searches will be screened for relevance in Endnote X9, and those identified as potentially eligible will be fully assessed against the inclusion/exclusion criteria. Two reviewers will independently double check the eligibility of the included studies and extract data by entering details into a predefined data acquisition form. The third researcher will check the article list and data extractions to ensure there were no duplicate articles or duplicate information of the same patient and resolved discrepancies regarding study inclusion. Furthermore, the references of the selected articles will be searched for articles that fit the criteria. Any lack of consensus will be adjudicated by the senior author. The authors will report reasons for exclusion of each study that will be chosen for full article review by means of the PRISMA flow diagram. A diagram of the study selection process is shown in Fig. 1.

#### 2.4.2 Data extraction

For articles meeting the inclusion criteria, data will be extracted from the eligible papers independently by 2 investigators. The data abstracted will include general characteristics (first author, year of publication, reference ID, etc), study characteristics (design of trial, control group, method of analysis, etc), participants (age, sex, country, etc), details of intervention (the names of CHM prescriptions for treatment, duration of treatment, and follow-up time, etc), outcome measures, etc. Discrepancies will be resolved by discussion or by the arbitration of a third investigator.

### 2.5 Outcome measures

The primary outcomes of this study included lung ventilation function, neurological manifestations associated with COVID-19, and all-cause mortality.

### 2.6 Risk of bias assessment

Two reviewers will independently assess the quality of the included studies according to the Cochrane collaboration's tool for RCTs and the Newcastle-Ottawa Scale (NOS) for cohort studies. The involved authors will be contacted if anything unclear. Any disagreements between the reviewers will be solved through discussion and consensus. Publication bias will be assessed using Egger test and funnel plots. *P*-value <.10 on Egger test will be considered statistically significant for publication bias. Inter-rater agreements between the researchers involved in study selection and those involved in the identification of the risk of bias will be assessed using *κ* Cohen coefficient.

### 2.7 Data synthesis and analysis

Data synthesis will be performed using the RevMan software (V.5.4). A random-effects model will be used to pool the data. Heterogeneity will be quantified in terms of Cochran *Q* statistics and *I*^2^ index. If significant heterogeneity was present (*P* values for *Q* statistic < .10), pooled estimates from random-effects models. Any discrepancy noticed in the process of data cross-checking will be resolved through discussion and the suggestion of a third reviewer.

The robustness of the main decisions made during the monitoring review process will be conducted through a sensitivity analysis. Several decision-making nodes for sensitivity review need to be considered in the system review process, such as methodological flaws, small research, and data loss. A sensitivity analysis will be implemented as suggested in the Cochrane Handbook. The results of the sensitivity analysis will be provided in the summary table. As shown by the results of the sensitivity analysis, the risk of bias will be discussed during the review process.

If the necessary data are available, subgroup analysis will be performed according to the type of control group. Variations will be considered in the characteristics of CHM prescriptions, dosage, and severity (ICU or Not ICU).

## 3 Ethics and dissemination

No human or animal subjects or samples will be used. The results will be published in a peer-reviewed journal, and will be disseminated at local and international neurology conferences.

## 4 Patient and public involvement

Patients were not involved in the development of the research question, outcome measures, or study design.

## Author contributions

**Conceptualization:** Yi-ming Sun, Ran Sun.

**Data curation:** Jia-yan Liu, Meng-lu Xiao.

**Formal analysis:** Jie Zhang, Gui-ping Li.

**Funding acquisition:** Gui-ping Li.

**Investigation:** Yi-ming Sun, Ran Sun.

**Methodology:** Gui-ping Li.

**Project administration:** Yi-ming Sun, Ran Sun.

**Software:** Jia-yan Liu, Jie Zhang.

**Supervision:** Gui-ping Li.

**Validation:** Gui-ping Li.

**Visualization:** Yi-ming Sun, Ran Sun.

**Writing – original draft:** Ran Sun, Meng-lu Xiao.

**Writing – review & editing:** Yi-ming Sun, Gui-ping Li.
